# Results of a cluster randomized trial testing the Systems Analysis and Improvement Approach to increase cervical cancer screening in family planning clinics in Mombasa County, Kenya

**DOI:** 10.1186/s13012-023-01322-y

**Published:** 2023-11-27

**Authors:** McKenna C. Eastment, George Wanje, Barbra A. Richardson, Emily Mwaringa, Shem Patta, Kenneth Sherr, Ruanne V. Barnabas, Kishorchandra Mandaliya, Walter Jaoko, R. Scott Mcclelland

**Affiliations:** 1 Departments of Medicine, University of Washington, Seattle, WA 98104 USA; 2 Global Health, University of Washington, Seattle, WA USA; 3 Biostatistics, University of Washington, Seattle, WA USA; 4 Vaccine and Infectious Disease Division, Fred Hutchinson Cancer Research Center, Seattle, WA USA; 5 Mombasa County Department of Health, Mombasa, Kenya; 6 Epidemiology, University of Washington, Seattle, WA USA; 7 Department of Medical Microbiology and Immunology, University of Nairobi, Nairobi, Kenya

## Abstract

**Background:**

Cervical cancer is the leading cause of cancer death in Kenyan women. Integrating cervical cancer screening into family planning (FP) clinics is a promising strategy to improve health for reproductive-aged women. The objective of this cluster randomized trial was to test the efficacy of an implementation strategy, the Systems Analysis and Improvement Approach (SAIA), as a tool to increase cervical cancer screening in FP clinics in Mombasa County, Kenya.

**Methods:**

Twenty FP clinics in Mombasa County were randomized 1:1 to SAIA versus usual procedures. SAIA has five steps: (1) cascade analysis tool to understand the cascade and identify inefficiencies, (2) sequential process flow mapping to identify bottlenecks, (3) develop and implement workflow modifications (micro-interventions) to address identified bottlenecks, (4) assess the micro-intervention in the cascade analysis tool, and (5) repeat the cycle. Prevalence ratios were calculated using Poisson regression with robust standard errors to compare the proportion of visits where women were screened for cervical cancer in SAIA clinics compared to control clinics.

**Results:**

In the primary intent-to-treat analysis in the last quarter of the trial, 2.5% (37/1507) of visits with eligible FP clients at intervention facilities included cervical cancer screening compared to 3.7% (66/1793) in control clinics (prevalence ratio [PR] 0.67, 95% *CI* 0.45–1.00). When adjusted for having at least one provider trained to perform cervical cancer screening at baseline, there was no significant difference between screening in intervention clinics compared to control clinics (adjusted PR 1.14, 95% *CI* 0.74–1.75).

**Conclusions:**

The primary analysis did not show an effect on cervical cancer screening. However, the COVID-19 pandemic and a healthcare worker strike likely impacted SAIA’s implementation with significant disruptions in FP care delivery during the trial. While SAIA’s data-informed decision-making and clinic-derived solutions are likely important, future work should directly study the mechanisms through which SAIA operates and the influence of contextual factors on implementation.

**Trial registration:**

ClinicalTrials.gov, NCT03514459. Registered on April 19, 2018.

Contributions to the literature

1. This rigorous cluster randomized trial tested an implementation strategy to improve cervical cancer screening in family planning clinics in Mombasa, Kenya.
2. The COVID-19 pandemic, a healthcare worker go-slow, and months-long strike disrupted FP clinic operations, likely impacting the results of the primary intent-to-treat analysis.
3. While the primary intent-to-treat analysis did not show an effect, data from the entire study period suggest that SAIA could be a useful strategy to improve cervical cancer screening in family planning clinics.
4. Data on the specific micro-interventions used by FP clinics highlight context-specific solutions to address bottlenecks in the cervical cancer screening care cascade.

## Introduction

The burden of cervical cancer is particularly high in sub-Saharan Africa, where the high prevalence of human immunodeficiency virus (HIV) infection contributes to cervical cancer incidence and severity [1, 2]. Cervical cancer can be prevented with early screening with visual inspection with acetic acid, cytology, and human papillomavirus (HPV) testing. Early treatment of abnormal cells or dysplastic lesions with cryotherapy, thermal ablation, and loop electrosurgical excision procedures can stop progression to cervical cancer [3–6]. Unfortunately, cervical cancer screening has been suboptimal in sub-Saharan Africa with screening uptake at 12.9% [7]. In Kenya, cervical cancer is the leading cause of cancer death in women, but a survey in 2014 found that only 14% of Kenyan women had ever been screened [8]. The Kenyan Ministry of Health has published guidelines that task family planning (FP) clinics and HIV clinics with providing cervical cancer screening [9].

Family planning clinics are a logical site to offer screening for reproductive-aged women who are presenting for family planning services, as they are sexually active and at risk for HPV infection, the leading cause of cervical cancer [10]. A survey of 70 FP clinics in Mombasa County conducted in 2017–2018 found that only 54% of clinic managers reported that their facility performed cervical cancer screening [11]. Public facilities and clinics with at least one provider trained to perform cervical cancer screening were more likely to report screening. Clinic managers identified the top three challenges to cervical cancer screening as lack of supplies to screen, lack of training, and clients declining the procedure.

Given the implementation gap between the proven effectiveness of cervical cancer screening and the integration of this evidenced-based intervention into routine care, this cluster randomized trial aimed to test whether a specific implementation strategy, the Systems Analysis and Improvement Approach (SAIA), was effective at increasing cervical cancer screening in FP clinics in Mombasa County, Kenya. SAIA is a multicomponent implementation strategy that uses plan-do-study-act cycles with a cascade analysis tool, sequential process flow mapping, and small tests of change (micro-interventions) to improve care cascades [12–15]. It was hypothesized that clinics randomized to SAIA would have a higher prevalence of cervical cancer screening compared to control clinics that received no intervention.

## Methods

### Study setting

This study was conducted in family planning clinics in Mombasa County, Kenya. FP clinics provide contraceptive services to male and female clients and are a source of primary care for reproductive aged individuals. At the start of the study, there were approximately 170 FP clinics in the county, including public and private facilities. All FP clinics receive commodities at no cost from the Mombasa County Department of Health Services (DOHS). Private clinics often charge a convenience fee for FP services including cervical cancer screening. All FP clinics are expected to follow local and national guidelines, which recommend that cervical cancer screening should be performed in FP clinics that are tier 2–tier 5 (dispensaries and private clinics, health centers, sub-county hospitals, county referral hospitals) when resources are available [16, 17]. Care in FP clinics is documented in large paper registry books that capture client demographics, commodities dispensed, and HIV counseling and testing services. In 2008, the FP register was updated to capture cervical cancer screening.

### Study design

This was a cluster randomized trial of 20 FP clinics in Mombasa County, Kenya, which was randomized 1:1 to an intervention arm implementing SAIA or a control arm following usual procedures.

### Participants

Family planning clinics were randomly selected from a representative sample of 70 clinics across all sub-counties that were surveyed as part of a landscape analysis [18]. Because this study was conducted in close cooperation with Mombasa County, any public or private FP clinic was eligible to participate in trial if their clinic managers provided assent for participation. Clinics that were expected to close during the study period and the 12 FP clinics that were involved in a previous SAIA trial were not eligible to participate [19]. After approval from the Mombasa County Department of Health, clinic managers were invited to participate in the cluster randomized trial. All clinic staff and managers were invited to participate in SAIA’s implementation and as participants in implementation of SAIA, making these frontline implementers and managers the “study subjects.” Staff from the research team supported clinic staff by facilitating sequential process flow mapping and then abstracting register data and inputting these data into the cascade analysis tool. The research staff also supported the FP clinic staff in idea generation for micro-interventions during monthly cycles and provided training for cervical cancer screening as one of the micro-interventions. The research team had no direct contact with FP clients. De-identified and aggregated data were collected from FP registers. FP clients were not identifiable and were not considered participants in this trial.

### Randomization

Randomization was performed by an independent statistician at the Center for AIDS Research Biometrics Core at the University of Washington. The statistician performing the randomization had no other role in implementing the trial. Restricted randomization was performed based on clinic size and whether any cervical cancer screening was being performed during the initial survey. Of the 70 clinics included in an initial survey in 2018, 20 were randomly selected for inclusion and randomized 1:1 to SAIA versus usual procedures. Twelve FP clinics that participated in a previous SAIA trial designed to increase HIV counseling and testing that were randomized to the SAIA intervention arm were not eligible to participate in this current study [19]. Because FP clinic staff participated in SAIA, blinding was not possible.

### Experimental implementation strategy versus control

This study evaluated SAIA, an evidenced-based five-step cycle composed of multiple implementation strategies [12–15]. The first step uses an Excel-based “cascade analysis” tool (CAT) to quantify the numbers of individuals who complete each cascade step and identify priority steps for improvement [20]. Step 2 involves sequential process flow mapping to identify modifiable bottlenecks in the system [13]. Step 3 develops and implements workflow modifications or small tests of change (micro-interventions) to address a bottleneck identified by front-line clinical providers in step 2. Step 4 assesses the impact of the micro-intervention and recalculates the CAT. Step 5 repeats the cycle [19]. SAIA maps to 13 Expert Recommendations for Implementing Change (ERIC) strategies: external facilitation, organization of provider implementation meetings, provision of ongoing consultation, facilitating relay of clinical data to providers, use of audit and feedback of routine data with healthcare teams, modeling and simulation of change, local needs assessment, local consensus discussions that include assessment of readiness and identification of barriers and facilitators, continuous quality improvement, development of a formal implementation blueprint, cyclical tests of change, and purposefully reexamining the implementation process [21].

After FP clinics were randomized, the clinic staff from SAIA facilities were invited for a full-day SAIA training that launched the trial. This training included facility-level flow mapping, discussion of each clinics’ cascade analysis using data from the CAT, and selection of the first micro-intervention. Cascade analysis and micro-intervention development, testing, and implementation were repeated monthly for 18 months from January 2020 to July 2021. Flow mapping was repeated as needed when clinic flow and processes had substantially changed. While the study staff supported the FP clinic staff in developing micro-interventions, the FP clinic staff were the implementers of their micro-interventions. FP clinics randomized to SAIA documented their monthly SAIA cycles on structured implementation plan posters provided by the research team. The posters included the CAT step targeted, barrier identified, and micro-intervention proposed. At the end of each monthly cycle, the clinic and research staff determined whether the micro-intervention was completed fully, partially, or not at all based on the tasks proposed and whether they were completed. The research staff photographed and transcribed these implementation plans to create a record of each SAIA cycle to categorize and track micro-interventions. Members of the research team (MCE and GW) categorized micro-interventions into categories that focused on the individual and health system levels. Where data were missing or questions arose, the staff examined the implementation plan photos to supplement the record. When transcribing the implementation plans, the study staff made notes about contextual factors for consideration when interpreting the data, such as clinic closure, staff on strike, or no FP clients seen during this interval.

The study staff conducted structured interviews with at least one staff member from each FP clinic in the trial to understand clinic and staff characteristics [11]. Data were captured on paper case report forms (CRFs) and entered into a REDCap database [22].

There was minimal interaction between the study staff and clinics randomized to the control arm. The study staff informed these FP clinics about the trial and its objectives, and the staff from control clinics participated in the structured interviews about clinic and staff characteristics. Outcome data from the FP registers was collected in control clinics on a quarterly basis.

Several data-cleaning steps were undertaken. Database entries were examined, and the FP register image was reviewed to determine whether data were available for missing responses. If so, these were added to the CRF and database. In addition, CRFs used to abstract FP register data were reviewed by a second study team member who independently viewed the digital FP register, verifying that the response on the CRF matched the data on the register image. Any discrepancies were resolved by discussion between data abstracters to arrive at a consensus about original register entries. Following data entry, the study staff performed a question-by-question assessment comparing hard copy CRFs to the digital database to identify and correct key-in errors.

### Outcomes

The primary outcome of this trial was the proportion of visits with eligible FP clients who were screened for cervical cancer using any approved method in clinics randomized to SAIA versus control clinics. A client was eligible for cervical cancer screening if they were 21–65 years old and were not current on recommended screening when counseled by FP providers.

### Power and sample size determination

Based on past data [11], it was assumed that FP clinics would see an average of 183 clients per 3-month period. It was also assumed that 8% of visits with eligible FP clients would include cervical cancer screening and that SAIA would increase the proportion of visits with cervical cancer screening of eligible clients to 20%. To calculate a sample size for a cluster RCT, the degree of similarity within a cluster (ρ) must be estimated, with literature often citing ρ≤ 0.25 and rarely exceeding 0.5 [23]. Conservatively, = 0.5 was used for power and sample size calculations, which translates to an intra-class correlation coefficient of approximately 0.05. The cluster size variability of FP clinics was calculated as 0.74. With *α* = 0.05, 10 clusters per arm were expected to provide 90% power to detect a difference of 12% in the prevalence of cervical cancer screening between SAIA clinics and control clinics.

### Statistical analysis

Counts/proportions and median (interquartile range) were calculated for baseline characteristics of SAIA clinics and control clinics. Counts/proportions were also used to describe micro-interventions that were fully, partially, or not implemented. Prevalence ratios were calculated using Poisson regression with robust standard errors to compare the effect of SAIA versus control conditions on the proportion of visits where eligible women were screened for cervical cancer during the last 3 months of the trial. The primary analysis followed the intent-to-treat principle. It was hypothesized that multiple cycles of SAIA might be needed to increase cervical cancer screening when some micro-interventions would not work, and this informed the decision to focus on the last 3 months of the trial. The unexpected COVID-19 pandemic initially restricted research activities as research teams were not permitted to visit the clinics. Also, routine procedures in healthcare clinics were disrupted when COVID-19 cases were high in Mombasa County. For these reasons, the study was extended by an additional 6 months beyond the initial 12 months planned for the trial.

Based on a previous analysis examining cervical cancer screening practices in FP clinics in Mombasa County, having at least one provider trained to perform cervical cancer screening was associated with this procedure being performed [11]. Baseline data highlighted differences between SAIA clinics and control clinics at study entry including the proportion of clinics that had at least one provider trained to perform cervical cancer screening. Best practices for cluster randomized trials often include adjustment for baseline differences in trial arms [24]. To control for these baseline differences, prevalence ratios were adjusted for having at least one provider trained to perform cervical cancer screening.

The national healthcare worker go-slow and strike that occurred in quarters three through five affected clinic operations and likely also impacted the primary results of the trial in quarter 6. To get a better sense of the effect of SAIA during the entire period of the trial, a post hoc secondary analysis was conducted using aggregated results from all 18 months of implementation.

### Ethical considerations

This trial is registered at ClinicalTrials.gov (NCT03514459) and was approved by the University of Washington Human Subjects Division and the University of Nairobi-Kenyatta National Hospital Ethics and Research Committee. The Mombasa County DOH endorsed this work as a county-supported public health activity, and clinic managers were asked to provide assent for their clinics’ participation. Clinic staff provided verbal assent to participate in structured interviews. The FP register data were fully de-identified, so consent was not required from individual FP clients.

## Results

**Fig. 1:**
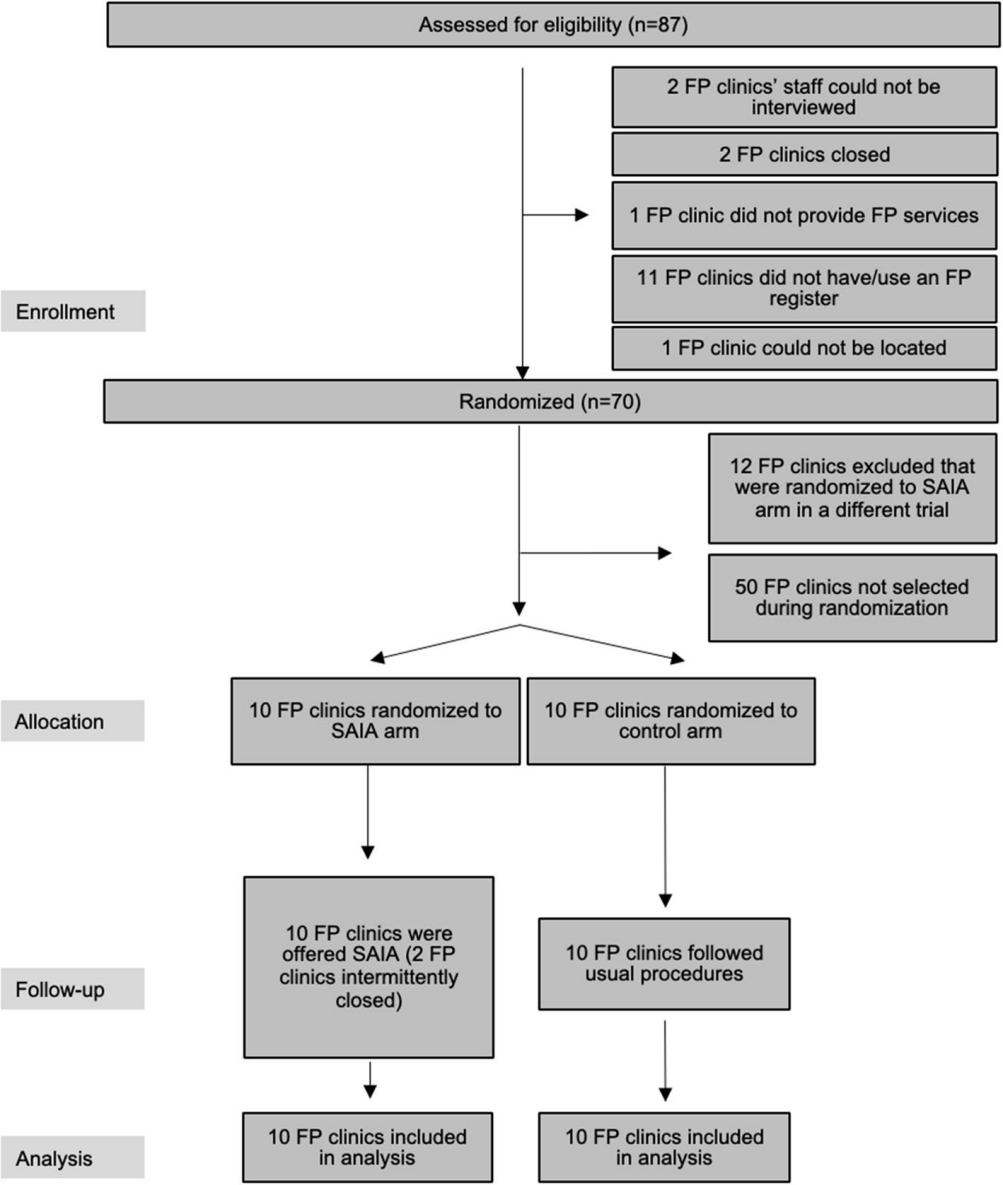
Flow diagram of FP clinics assessed for eligibility, randomized, participated, and included in final analyses

From the 70 clinics in the cross-sectional survey, 20 were randomly selected for participation in the trial. These clinics were randomized 1:1 to the SAIA and control arms (Fig. 1). Of the clinics randomized to SAIA, one was intermittently closed, but all ten were included in the analysis. Eighty percent (*n* = 8) of SAIA clinics and 50% (*n* = 5) of control clinics were public facilities, and 70% (*n* = 7) of SAIA clinics and 40% (*n* = 4) of control clinics were in urban locations (Table 1). Clinics randomized to SAIA had a median of five (interquartile range [IQR] 4–7) providers compared to four (2–5) providers in control clinics. Thirty percent (*n* = 3) of SAIA clinics reported that they performed cervical cancer screening at baseline compared to 60% (*n* = 6) of control clinics. Of the three SAIA clinics that provided cervical cancer screening, 66% (2/3) charged a convenience fee for screening. Among the six control clinics that provided cervical cancer screening at baseline, 33% (2/6) charged a fee. Clinics randomized to SAIA had a median of zero providers (*IQR* 0–1) trained to perform cervical cancer screening compared to two providers (*IQR* 1–3) trained in control clinics.

**Table 1:** Baseline characteristics of family planning clinics randomized to SAIA arm and control arm

	**SAIA clinics** ***N* = 10** ***n* (%) or median (IQR)**	**Control clinic** ***N* = 10** ***n* (%) or median (IQR)**
Public clinic	8 (80)	5 (50)
Urban clinic	7 (70)	4 (40)
Number of FP provider staff	5 (4–7)	4 (2–5)
Any cervical cancer screening done at baseline	3 (30)	6 (60)
Fee required for cervical cancer screening	2/3 (66%)	2/6 (33%)
At least one provider trained in cervical cancer screening	4 (40)	8 (80)

*Abbreviations*: *IQR* Interquartile range, *FP* Family planning

**Fig. 2:**
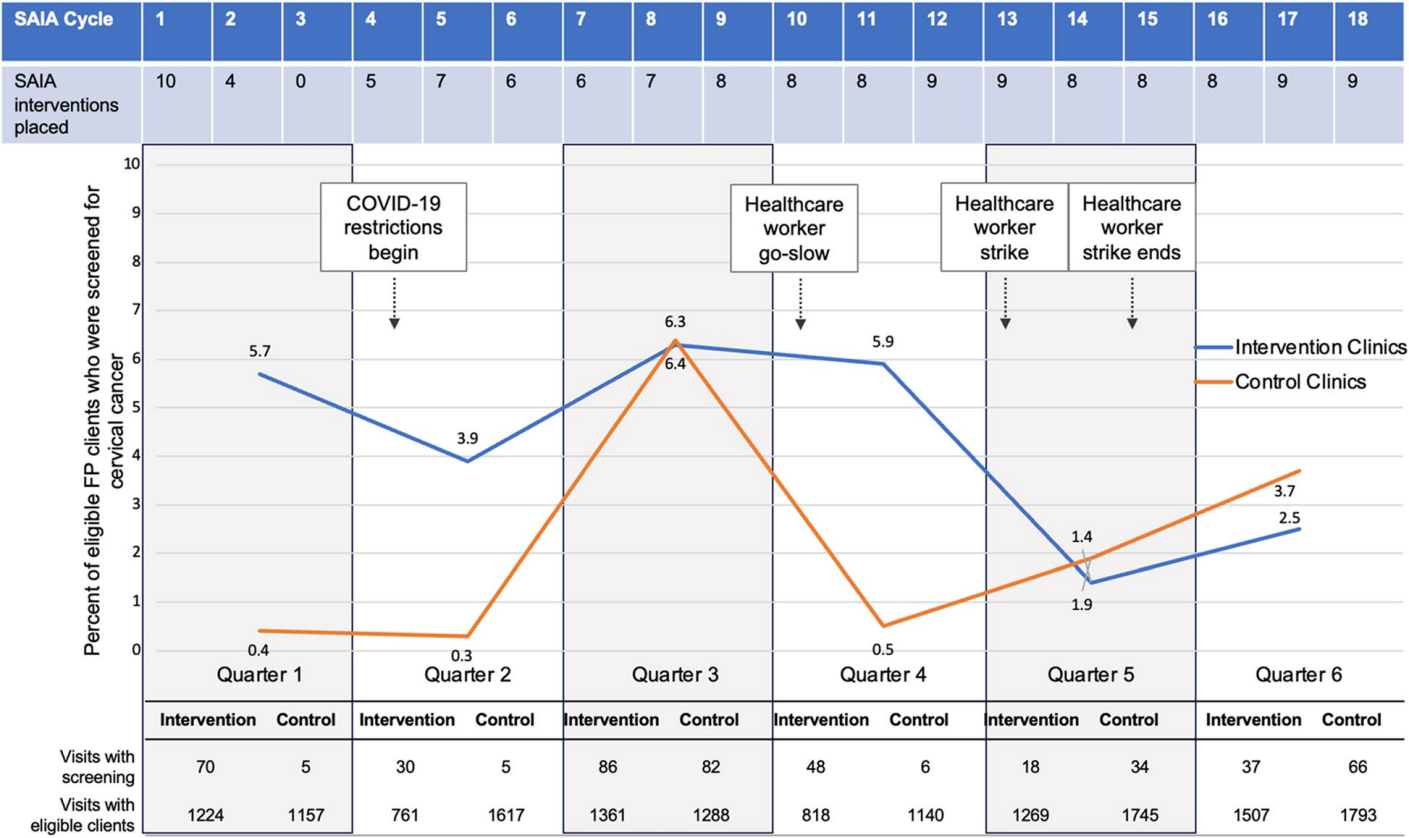
Percent of visits with eligible FP clients with cervical cancer screening in SAIA clinics compared to control clinics with associated SAIA cycles and SAIA interventions placed

In the last quarter of the trial, 2.5% (37/1507) of visits with eligible FP clients at SAIA facilities included cervical cancer screening compared to 3.7% (66/1793) in control clinics (prevalence ratio [PR] 0.67, 95% confidence interval [CI] 0.45–1.00) (Table 2). When adjusted for having at least one provider trained to perform cervical cancer screening at baseline, there was no significant difference between screening in SAIA facilities compared to control clinics in the last quarter of the trial (adjusted *PR* 1.14, 95% *CI* 0.74–1.75). One SAIA clinic closed in month 2 and remained closed for the duration of the trial. Figure 2 depicts the percentage of eligible FP clients who were screened for cervical cancer in SAIA clinics and control clinics across the six quarters of the trial. The timing of the onset of COVID-19 restrictions, the healthcare worker go-slow, and the healthcare worker strike are shown. The absolute numbers of clients screened and the total number of clients seen in each quarter of the trial are presented.

**Table 2:** Prevalence ratios of visits with eligible family planning clients where cervical cancer screening occurred in SAIA clinics compared to control clinics

**Quarter**	**Proportion of visits where eligible FP clients were screened for cervical cancer in SAIA clinics**	**SAIA interventions placed in intervention clinics out of the total possible interventions if completed monthly**	**Proportion of visits where eligible FP clients were screened for cervical cancer in control clinics**	**Prevalence ratios (95% confidence intervals)**	**Adjusted prevalence ratios (95% confidence intervals)**^a^
January 2020–June 2021	289/6940 (4.2%)	108/180	198/8740 (2.3%)	1.84 (1.54–2.20)	2.27 (1.86–2.77)
1: January 2020–March 2020	70/1224 (5.7%)	22/30	5/1157 (0.4%)	13.23 (5.34–32.7)	11.60 (4.56–29.52)
2: April 2020–June 2020	30/761 (3.9%)	22/30	5/1617 (0.3%)	6.80 (2.49–18.56)	10.65 (3.90–29.07)
3: July 2020–September 2020	86/1361 (6.3%)	23/30	82/1288 (6.4%)	0.99 (0.73–1.34)	1.37 (0.99–1.91)
4: October 2020–December 2020	48/818 (5.9%)	18/30	6/1140 (0.5%)	11.15 (4.77–26.05)	11.69 (4.59–29.7)
5: January 2021–March 2021	18/1269 (1.4%)	22/30	34/1745 (1.9%)	0.73 (0.41–1.29)	1.42 (0.80–2.51)
6: April 2021–June 2021	37/1507 (2.5%)	22/30	66/1793 (3.7%)	0.67 (0.45–1.00)	1.14 (0.74–1.75)

*Abbreviations*: *SAIA* Systems Analysis and Improvement Approach

^a^Adjusted for having at least one provider trained to perform cervical cancer screening

In a post hoc secondary analysis including 18 months of the trial, 4.2% (289/6940) of visits with eligible FP clients at SAIA clinics compared to 2.3% (198/8740) of visits in control clinics included cervical cancer screening (PR 1.84, 95% *CI* 1.54–2.20). This association was stronger after adjustment for baseline provider training for cervical cancer screening (*aPR* 2.27, 95% *CI* 1.86–2.77). A post hoc power calculation showed that the secondary analysis had > 99% power to detect this difference in prevalence.

Clinics randomized to SAIA had SAIA micro-interventions placed in 129 of 180 possible months. The micro-interventions were categorized into client-level and clinic-level interventions (Table 3). More than half of micro-interventions involved sensitization of clients (56%, 72), and interventions to mobilize clients occurred in 3% (4) of SAIA cycles. Clinic-level micro-interventions included addressing lack of commodities (26%, 34), training in cervical cancer screening (8%, 10), restructuring of clinic flow (2%, 2), improving documentation (2%, 2), making renovations to facilitate cervical cancer screening (2%, 2), and addressing the cost of cervical cancer screening for clients (1%, 1). Out of the 129 micro-interventions proposed, 44% (57) were implemented fully, 31% (40) were implemented partially, and 25% (32) were not implemented at all. There were no adverse events from participating in this trial.

**Table 3:** Categorization of micro-interventions in SAIA clinics

Types of micro-interventions	Proportion of SAIA cycles that addressed this micro-intervention (*n* = 129)	Examples of specific micro-interventions	Notes
Client level			
Sensitization of clients	72 (56)	Health talks	Client sensitization on importance of cervical cancer screening led one clinic to screen all eligible clients in one month
Mobilizing clients	4 (3)	Using community health volunteers to mobilize clients to come in for screening	One clinic mobilized clients for screening and in 1 month screened all eligible clients
Clinic level			
Addressing lack of commodities for screening	34 (26)	Outsource commodities	One clinic was able to meet with management to procure screening supplies
Training for cervical cancer screening	10 (8)	Cervical cancer training conducted by study staff	In one clinic, following cervical cancer screening training, 34 of 83 eligible clients in a month were screened
Clinic flow restructuring	2 (2)	Conduct cervical cancer screening with FP services on days when clinic is not usually busy	One clinic was providing FP clinic in a building constructed from a shipping container that became very warm throughout the day. The clinic restructured to have FP clinic in the morning before temperatures in the building was too warm
Improve documentation	2 (2)	Ensure completeness of register	One clinic provided feedback about register documentation and also performed data cleaning to improve quality of documentation
Clinic building renovation	2 (2)	Moving FP clinic into a container onsite	Extra space for cervical cancer screening was made available in a clinic undergoing building renovation
Addressing cost of cervical cancer screening	1 (1)	Subsidize cost of cervical cancer screening	Clinic subsidized cost of screening and proportion of eligible FP clients screened went from 3 to 13%
Missing data on specific micro-interventions	2 (2)		

## Discussion

In the primary intent-to-treat analysis, SAIA was not associated with a significant difference in visits where women were screened for cervical cancer in SAIA versus control clinics in the last quarter of the trial. Because the COVID-19 pandemic and healthcare worker strike impacted routine clinic operations in SAIA and control clinics, the pre-specified primary analysis may not be the best reflection of SAIA’s impact in this trial. When results were aggregated across 18 months, there is preliminary evidence that SAIA was associated with a nearly twofold increase in the proportion of visits with cervical cancer screening compared to controls, offering preliminary evidence.

Integrating cervical cancer screening into FP clinics is a promising implementation strategy, particularly in low-and middle-income countries (LMICs), which can lower the burden on clients for additional visits and could increase the efficiency of resources being used [25]. A study conducted in Kenya highlighted that women prefer breast and cervical cancer screening to be integrated into routine care [26]. This is also supported by a framework for cervical cancer elimination in LMICs that supports integrating care for cervical cancer screening into antenatal care, HIV clinics, and FP clinics [27].

While the integration of services is one promising implementation strategy for increasing cervical cancer screening, other implementation strategies have also been evaluated. A scoping review in 2020 mapped different implementation strategies to improve cervical cancer screening in sub-Saharan Africa [28]. Of the 19 studies included in the review, more than half tested education-based interventions targeted to potential cervical cancer screening clients. In general, these interventions were not very effective. Studies testing interventions utilizing community health educators and peer educators had more success. Innovative service delivery models including community health worker outreach and enhanced counseling were also associated with increases in cervical cancer screening. A different systematic review also concluded that while education-based strategies were common, these were not the most effective strategies [29]. This review stressed the need for new implementation strategies. The different context-specific micro-interventions that FP clinics implemented during the trial could be categorized as adapting service delivery within the FP clinic, education of providers and clients, and improved counseling to clients by healthcare providers. These newer implementation strategies may explain some of SAIA’s impact on cervical cancer screening.

To date, SAIA has been tested in other care cascades including preventing-mother-to-child transmission (PMTCT) in Kenya, Mozambique, and Cote d’Ivoire, HIV counseling and testing in FP clinics in Kenya, mental health in Mozambique, and the pediatric HIV cascade in Kenya. When used to improve the PMTCT cascade, SAIA was associated with improvements in antiretroviral coverage and the proportion of HIV-exposed infants screened for HIV in pre-specified sub-group analyses, though these associations were not statistically significant. HIV testing in antenatal clinics was not different in clinics implementing SAIA compared to usual procedures [12]. In FP clinics in Mombasa County, Kenya, SAIA was associated with significantly more HIV counseling and testing compared to usual procedures in a cluster randomized trial of 24 clinics [19]. These results were maintained after transitioning SAIA’s facilitation to Mombasa County Department of Health staff [30]. In the mental healthcare cascade in Mozambique, SAIA was associated with higher odds of on-time follow-up for appointments, adherence to medications, and functional improvement but was not associated with initial mental health diagnosis or medication selection, enrollment in follow-up care, or return to care after initial mental health consultation [31]. When tested to improve the pediatric and adolescent HIV care cascade, SAIA was significantly associated with an increase in ordered viral load tests but was not associated with more HIV testing, linkage to care, or viral load suppression in a pre-post analysis [32]. In these prior implementations of SAIA, results have ranged from improvements in one step of a care cascade to significant improvements in the entire cascade. While endpoint results have been mixed, variability may be explained by the range of barriers and bottlenecks present in different steps in these varied cascades across different countries.

In this present study when SAIA was tested to improve cervical cancer screening, a number of unique factors contributed to the lack of significant effect of the primary analysis. This study occurred concomitantly with the go-slow and strike and the COVID-19 pandemic, both of which unexpectedly and profoundly disrupted healthcare delivery across Mombasa. One SAIA clinic closed in month 2 of the trial. In other clinics, the pandemic restrictions and healthcare worker strike disrupted operations. This may explain why less than half of the proposed micro-interventions were implemented fully. Cervical cancer screening declined sharply in both SAIA and control clinics during the strike, highlighting the impact of healthcare disruptions on necessary preventative care. The primary analysis was scheduled for the end of the trial period, to allow time for SAIA’s effect to have the greatest impact over the 18-month study period, but this coincided with the end of the months-long healthcare worker go-slow and strike. Our experience with this trial and previously with HIV counseling and testing in FP clinics highlighted that SAIA’s effects were observed soon after the intervention and generally persisted over time with the exception of the major contextual impacts detailed previously. In this context, we propose that future SAIA trials should consider including data from the full implementation period in their primary analyses.

This study had several strengths. First, the cluster randomized trial design rigorously tested SAIA in the cervical cancer screening care cascade. Second, this research included a collection of details about specific micro-interventions that clinics proposed and implemented. Granular data on the micro-interventions selected, how well they were implemented, and the effect on cervical cancer screening provided a better understanding of how SAIA worked. Finally, the quality control procedures employed in this study helped to optimize the capture and quality of the programmatic data examined to identify study outcomes.

A few limitations are important to note. These data present the prevalence of visits where cervical cancer screening was performed rather than the proportion of eligible women who were screened. To measure the proportion of eligible women screened, there would need to be a robust system for following individual FP clients over time. This was not possible with the paper registers in use at the time of the study. Despite this limitation, a post hoc secondary analysis of data for 18 months of implementation was able to detect an overall effect of the SAIA implementation strategy on the proportion of visits at which cervical cancer screening was performed. This secondary analysis had > 99% power in a post hoc power calculation. Next, despite randomization, the relatively small number of clinics in the trial contributed to baseline differences between the SAIA arm and the control arms, particularly in provider training for cervical cancer screening. This limitation was mitigated by performing analyses that adjusted for baseline differences [24]. Lastly, this implementation study used routinely collected program data, and clinicians with busy workloads may not have time to document fully in the FP registers. As a result, it can be difficult to know the extent to which improved rates of cervical cancer screening were related to better documentation versus actual increases in the proportion of visits at which screening was performed. In this context, it is notable that few of the micro-interventions in the SAIA arm of the trial focused on improving documentation.

## Conclusion

In conclusion, this cluster randomized trial tested SAIA as a tool to improve cervical cancer screening. Although the primary intent-to-treat analysis did not show an effect, data from 18 months of the randomized trial provide preliminary evidence that SAIA could be a useful strategy to improve rates of cervical cancer screening in FP clinics. While SAIA’s data-informed decision-making and clinic-derived solutions that incorporate a variety of implementation strategies may be important, future work should directly evaluate the mechanisms through which SAIA operates and the influence of contextual factors on implementation.

## Data Availability

This study was conducted with approval from the Kenyatta National Hospital—University of Nairobi Ethics and Research Committee (KNH-UON ERC), which requires that we release data from Kenyan studies (including de-identified data) only after they have provided their written approval for additional analyses. As such, data for this study will be available from the authors upon request, with written approval for the proposed analysis from the KNH/UON ERC. Their application forms and guidelines can be accessed at https://erc.uonbi.ac.ke/. Please contact the principal of the KNH-UON ERC at principal-cae@uonbi.ac.ke.
